# Long Intergenic Noncoding RNA 00641 Promotes Growth and Invasion of Colorectal Cancer through Regulating miR-450b-5p/GOLPH3 Axis

**DOI:** 10.1155/2022/8259135

**Published:** 2022-06-15

**Authors:** Zhongshi Hong, Jianpeng Pan, Mingliang Chen, Xian Deng, Zhichuan Chen, Chunxiao Wang, Chengzhi Qiu

**Affiliations:** Department of General Surgery, The Second Affiliated Hospital of Fujian Medical University, Quanzhou, Fujian 362000, China

## Abstract

**Background:**

Long noncoding RNAs (lncRNAs) have a vital function in tumor onset and progress. For instance, long intergenic noncoding RNA 00641 (LINC00641) has been linked to cancer modulation. Nonetheless, the precise biological roles of LINC00641 in colorectal cancer (CRC) remain elusive.

**Methods:**

The expression levels of LINC00641 as well as the docking sites for LINC00641 and miR-450b-5p were analyzed using public data resources and web-based analytic tools. The putative downstream targets of miR-450b-5p were also predicted. Next, we evaluated the biological functions and the contents of LINC00641 in CRC both *in vivo* and *in vitro*. We next explored the influence of LINC00641 on the growth, migration, and infiltration of CRC cells via cell proliferation, migration, and invasion experiments. Besides, qRT-PCR, western blotting, flow cytometry, luciferase enzyme reporter assay, and in vivo tumorigenicity assays were conducted.

**Results:**

Our results confirmed that LINC00641 was markedly upmodulated in CRC tissues and CRC cell lines, and the upmodulation was linked to poor survival. Notably, the proliferative and migratory abilities of HCT-116 and SW480 cells were significantly inhibited by the knockdown of LINC00641 both *in vitro* and *in vivo*, illustrating that LINC00641 exerted a tumor-promotion role in CRC. Mechanistically, LINC00641 could competitively bind miR-450b-5p, thereby expunging its inhibitory effect on GOLPH3 expression. Moreover, miR-450-5p and GOLPH3 were able to reverse LINC00641-mediated cellular processes.

**Conclusions:**

Overall, the findings of this study suggest that LINC00641 promotes the proliferative and migratory abilities of CRC through sponging the miR-450b-5p/GOLPH3 axis.

## 1. Introduction

Among cancer-related deaths globally, colon cancer (CRC) is the third most common type which ranks second among cancer-related deaths [1]. It is worth noting that CRC development is a multistep process involving various oncogenes and tumor suppressor genes. Specifically, the process involves protein-coding genes, such as P53, APC, RAS, and GOLPH3, and other types of noncoding RNAs. The interaction between these genes regulates downstream protein expression and affects CRC's progression, metastasis, and chemoresistance. However, the distinct underlying mechanisms of the regulation have not yet been fully elucidated. Therefore, this necessitates a greater knowledge of the mechanisms behind CRC formation and progression, with the overarching goal of generating more effective treatments and finding novel ways for early detection.

Long intergenic noncoding RNAs (lncRNAs) constitute a class of RNA transcripts with more than 200 nucleotides but cannot be converted into proteins [2]. Emerging evidence has revealed that lncRNAs harbor vital modulatory role among numerous biological processes, including cancer. In addition, recent studies have discovered that lncRNAs regulate numerous cellular processes in CRC, for instance cell cycle progress, apoptosis, invasion, and metastasis [3–5]. And they have been documented to participate in the modulation of cancer stem-like cells (CSC) and epithelial-mesenchymal transition (EMT) phenotypes in CRC [6, 7]. Among the various lncRNAs, a novel lncRNA termed as long intergenic noncoding RNA 00641 (LINC00641) has attracted our attention. LINC00641 was first discovered by Liang et al. [8] as a disease-associated lncRNA. It is located at 14q11.2 in the chromosome and has been documented to harbor an oncogenic role and a tumor-repressive function in various human cancers. For example, LINC00641 is involved in regulating the onset and progress of non-small-cell lung cancer [9], renal cell carcinoma [10], and gastric adenocarcinoma [11]. Nonetheless, the underlying mechanisms of LINC00641 in CRC involve a complex process, which remains unclear to date.

LncRNAs modulate gene expression at multiple stages, such as the levels of transcription, pre-transcription, and post-transcription, to modulate numerous biological processes. Previous research evidence confirms that Golgi phosphoprotein 3 (GOLPH3) is the first Golgi resident oncoprotein and drives cancer through binding tightly at the *trans*-Golgi to PtdIns(4)P [12, 13]. In our previous investigations, we established that the overexpression of GOLPH3 promotes the growth, migration, and chemoresistance of colon cancer cells by activating multiple intracellular signal pathways [14–16]. Besides, elevated GOLPH3 expression has been linked with a poor outcome in colon cancer, [17] and is a possible predictor of 5-FU chemosensitivity [18]. However, only a handful of investigations have explored the upstream regulation of GOLPH3 expression. In the recent times, it has been documented that noncoding RNAs, for instance miR-126, miR-3150b, and miR-3150b-3p may downmodulate GOLPH3 expression and decrease growth by interacting with the promoter in many malignancies [19–21]. However, there is little knowledge on the regulation of GOLPH3 expression by lncRNAs in CRC. The main purpose of this research work was to assess the expression and precise role of LINC00641. starBase postulated that LINC00641 may target miR-450b-5p. We established that the contents of miR-450b-5p were downmodulated in recurrent CRC tissues. It has been documented that miR-450b-5p dampens stemness and 5-FU chemoresistance development in CRC via targeting SOX2 [22]. Mechanically, LINC00641 might compete with miR-450b-5p, leading to the upregulation of its targets N-cadherin, SOX2, and Twist that are involved in EMT and CSC regulation. In addition, we explored the potential underlying mechanism to verify whether LINC00641 can upregulate GOLPH3 expression through sponging miR-450b-5p, ultimately exerting an oncogene function in colorectal cancer.

## 2. Materials and Methods

### 2.1. Cell Lines

Primary CRC cell lines (SW480, HT-29, and HCT-116), metastatic CRC cell line SW620, NCM460 normal colon mucosal epithelial cell line, and 293T cell line were provided by Xiamen Immocell Biotechnology Co., Ltd (Xiamen, Fujian, China. URL: http://immocell.com/html/cn/pc/cn_about.html). All the CRC cells, and NCM460 cells were inoculated in DMEM (catalog number: D6429, Gibco, Detroit, MI, USA) augmented with 10% FBS (Gibco) under 37°C and 5% CO_2_ conditions.

### 2.2. Database Analysis

A dataset GSE33113 with clinical-pathological parameters of 90 CRC patients' and 6 healthy donors' tissues were abstracted from the Gene Expression Omnibus (GEO) data resource (https://www.ncbi.nlm.nih.gov/geo/) and then used to analyze the level of LINC00641. The exploratory cohort comprising of The Cancer Genome Atlas (TCGA) cases were abstracted from the GDC data resource (https://portal.gdc.cancer.gov) and UCSC Xena data resource (https://tcga.xenahubs.net). Next, we standardized and compared the data of LINC00641 expression levels in CRC tissue; if the obtained value was greater within CRC tissue than healthy tissue, the expression would be regarded as “high expression.” Otherwise, it was “low-expression.” The overall survival (OS) and disease-free interval survival (DFIS) rates in CRC subjects were subjected to Kaplan–Meier analysis. The patients' information was abstracted from the public data resource. Notably, this research premise was exempted from ethical approval by the database administrators due to its retrospective nature. Datasets GSE81581 and GSE from the GEO database were used to screen downregulated miRNAs and upregulated mRNAs, respectively. Differentially expressed miRNAs or mRNAs between nonmalignant and CRC tissues were identified by R (version 4.0.5) package limma (version 3.44.3) based on a false discovery rate (FDR) cutoff <0.05 and a |log2fold change| cutoff >1.

### 2.3. qRT-PCR

The isolation of total RNA from CRC cell lines or xenograft tumors was done with the TRIzol reagent (Invitrogen, Carlsbad, United States, CAT#15596026) as described by the manufacturer. Thereafter, the generation of cDNA was done with the TaqMan® MicroRNA Reverse Transcription Kit (Invitrogen; CAT#4366596). qRT-PCR was run on the iQ5 Real-Time PCR Platform (Bio-Rad Laboratories, Hercules, CA, United States) with a ChamQ SYBR qPCR Master Mix (Vazyme, Nanjing, China; CAT#Q311-02). The thermocycling settings were as follows: 95°C for 30 s, followed by 40 cycles of 95°C for 5 s, and 60°C for 15 s. Relative gene expression level was normalized to 18S RNA (for mRNAs) or snRNA U6 (for miRNAs) and computed using the 2^−ΔΔCt^ approach.  Table S1 provides the list of primers used.

### 2.4. Plasmid Construction

The isolation of total RNA from HCT-116 cells was done with the RNA Isolater Total RNA Extraction Reagent (CAT#R401-01, Vazyme). The binding site of miR-450-5p at wild-type LINC00641 (LINC00641 WT) was amplified from total RNA using PrimeScript II (CAT#6210A, Takara, Tokyo-to, Japan) and cloned in the pmirGLO vector (CAT#E1330, Promega, Madison, United States), downstream of the firefly luciferase reporter gene. The wild-type 3′UTR of the GOLPH3 gene (GOLPH3 3′UTR-WT) was PCR amplified from the genomic DNA of HCT-116. The GOLPH3 3′UTR fragment was also cloned into the pmirGLO vector, downstream of the firefly luciferase reporter gene. The generation of the mutant versions of pmirGLO-LINC00641 WT and pmirGLO-GOLPH3 3′UTR (pmirGLO-LINC00641 MUT and pmirGLO-GOLPH3 3′UTR MUT) was done with the QuikChange II Site-Directed Mutagenesis Kit (Agilent Technologies, United States) as documented by the manufacturer Lentiviral plasmid pLKO.1-TRC-MCS-T2A-puro (Antihela, Xiamen, Fujian, China) was utilized for knockdown of LINC00641 plasmid. Notably, hsa-miR-450b-5p mimic and mimic NC were supplied by Ribobio (Guangzhou, China). Thereafter, the cDNA of GOLPH3 was reverse transcribed from total RNA using PrimeScript II and inserted into the overexpression vector pCDH-EF1a-MCS-T2A-bsd (Antihela).  Table S2 provides the primer sequences utilized for plasmid construction.

### 2.5. Lentivirus Generation and Infection

We maintained the 293T cells in a 10-cm dish. Exfect Transfection Reagent was utilized to co-transfect the cells with pLKO.1-shLINC00641 or vector plasmid (12 *μ*g), psPAX2 (8 *μ*g, Antihela), and pMD2.G (4 *μ*g, Antihela) when they attained 80% confluence (Vazyme). Fresh medium was introduced after 4 hours to create lentiviruses for another 48 hours. Thereafter, we collected the lentivirus supernatant and utilized it to infect cells at a multiplicity of infection (MOI) of 40 with 10 g/mL polybrene (CAT#40804ES76, Yeasen, Shanghai, China), thus creating stable LINC00641-silenced HCT-116 cells or control cells.

### 2.6. MTT Assay

The viability of cells was explored via the MTT assay kit (CAT#40206ES76, Yeasen) as described by the vendor. Concisely, cells were planted and transfected in a 96-well plate. Cells were then stained with MTT at different time points (0 h, 24 h, 48 h, and 72 h), and the optical density of every sample at 490 nm was assessed using a SpectraMax Absorbance Reader (Molecular Devices, San Francisco, United States).

### 2.7. Cell Cycle Assay

Cells were first fixed at 4°C in 70% ethanol overnight. Afterwards, the fixed cells were inoculated for 30 mins with 0.5 mL PBS enriched with 10 *μ*g/mL RNase (CAT#B694345-0025, Sangon Biotech, Shanghai, China) and 0.2% Triton X-100 (CAT#A110694-0100, Sangon Biotech) at 37°C, followed by staining with 20 *μ*g/mL PI (CAT#A211-02, Vazyme) for 30 min at 28°C. Finally, cells were analyzed with the flow cytometer NovoCyte 1300 Platform (ACEA, San Diego, USA).

### 2.8. Apoptosis Assay

Staining of cells was done for 10 min with 5 *μ*L FITC and 5 *μ*L PI (CAT#A211-02, Vazyme) at 28°C as described by the manufacturer. Next, the stained cells were analyzed with a NovoCyte 1300 flow cytometer, and 1 × 10^4^ cells were analyzed using the FITC-channel (Ex: 488 nm/Em: 519 nm) and PE-channel (Ex: 488 nm/Em: 578 nm).

### 2.9. Transwell Migration and Invasion Assays

Transwell plates were utilized to perform transwell migration and invasion assays (CAT#3422, Corning, Corning, United States) without Matrigel (CAT#356234, BD Biosciences, Sparks, Ubited sates) for migration assays or with Matrigel for invasion assays. Briefly, we suspended the cells with FBS-free DMEM at a density of 5 × 10^5^/mL, followed by the addition of 100 *μ*L cell suspension to the upper compartment and 500 *μ*L of DMEM enriched with 10% FBS to the lower compartment. After 24 h, the migrated and invasive cells were stained with 0.2% crystal and then photographs were captured with a light microscope MOTIC AE31 (MOTIC, Hongkong, China). Finally, cell numbers were counted with the ImageJ 1.52v (NIH, Bethesda, MD, USA).

### 2.10. Dual-Luciferase Reporter Assay

Binding sites of miR-450b-5p at LINC00641 or GOLPH3 3′UTR were predicted using an online database, which was used for generating luciferase vectors: pmirGLO-linc00641-WT/MUT and GOLPH3 3′UTR-WT/MUT. MiR-450b-5p was co-transfected with the luciferase vectors, consisting of pmirGLO-linc00641-WT/Mut or GOLPH3 3′UTR-WT/MUT luciferase plasmids. After 24 h of post-transfection, the Dual-Luciferase Assay System (CAT#E1910, Promega) was adopted to assess the luciferase enzyme activity as described by the manufacturer.

### 2.11. Western Blotting Analysis

Cells and tissue homogenates were lysed in RIPA lysis buffer (CAT#P0013 B, Beyotime, China), and then the quantification of the total protein was performed with the BCA protein concentration determination kit (CAT#P0012S, Beyotime). Protein samples (12 *μ*g/lane) were then resolved using on 10% SDS-PAGE gel electrophoresis and blotted to PDVF membranes (CAT#IPVH00010, Millipore, MA, USA). Afterwards, membranes were inoculated overnight with the primary antibodies, including Anti-GOLPH3 (1 : 1000, CAT#191121-1-AP, Proteintech, Wuhan, Hubei, China) and Anti-GAPDH (1 : 2000, CAT#10494-1-AP, Proteintech) at 4°C. Thereafter, we washed the membranes and then inoculated them for 1 hour with HRP-linked goat anti-rabbit IgG (H + L) (1 : 2000, CAT#SA00001-2, Proteintech) at 28°C. Then, visualization of bands was performed with the chemiluminescence detection reagent (CAT#WP20005, Thermo Fisher Scientific, Carlsbad, United States) and semi-quantified through densitometry with the ImageJ 1.52v.

### 2.12. Xenograft Tumor Model

BALB/c-nude mice (6–8 weeks old) from Shanghai SLAC Laboratory Animal Co., Ltd were used in the xenograft tumor model. For orthotopic assays, we subcutaneously inoculated 5 × 10^6^ cells into the lower back regions of the mice (*n* = 6 per group). Thereafter, we euthanized the mice 32 days post-orthotopic inoculation, and the growth of subcutaneous tumors was quantified. Weighing of the tumor tissues was performed, followed by grinding with grinder Tissuelyser-24 (Jingxin, Shanghai, China). Thereafter, qRT-PCR, western blotting analysis, and IHC assay were used to assay the homogenates.

### 2.13. Immunohistochemical Assay

The deparaffinized and rehydrated tissue slide was pretreated for 25 min with peroxidase blocking buffer (CAT#LS-M24-100, LSBio, Seattle, WA, USA) at 28°C. Antigens were retrieved using citrate buffer (pH 6.0) at 28°C for 30 min. Afterwards, we inoculated the tissue slides for 1 hour with 5% BSA in PBS at 28°C. Subsequently, the slides were overnight inoculated with Anti-Ki67 antibody (CAT#9027T, Boston,MA, USA) at 4°C. Thereafter, the tissue chip was inoculated for 30 min with biotin-linked secondary antibody (CAT#KIT-7710, MXB Biotechnologies, Fuzhou, Fujian, China) at 28°C, followed by inoculation for 15 min with HRP-linked streptavidin (CAT#KIT-7710, MXB Biotechnologies) at 28°C. Finally, Ki67 signal was determined by DAB staining (CAT#DAB0031, MXB Biotechnologies). The images were captured with a panoramic scanner PANNORAMIC (3DHISTECH, Budapest, Hungary) using the software CaseViewer 2.4 (3DHISTECH). The integrated optical density (IOD) and Ki67+ area were analyzed with the software AIpathwell (Servicebio, Wuhan, China). Mean density of Ki67 was calculated using the formula: mean density = IOD/Ki67+ area.

### 2.14. Statistical Analysis

All statistical analyses were implemented in GraphPad Prism 7 (GraphPad Software, San Diego, CA, USA), and all experimental data are presented as mean ± standard deviation. One-way analysis of variance (ANOVA), followed by Tukey's post hoc test was utilized for multiple comparisons among three groups, whereas Student's *t*-test was done to compare the difference between two groups. The generation of survival curves was done with the Kaplan–Meier approach, and the significance was assessed via the log-rank test. *P* < 0.05 denoted statistical significance.

## 3. Results

### 3.1. LINC00641 Was Upregulated in CRC

**Figure 1:**
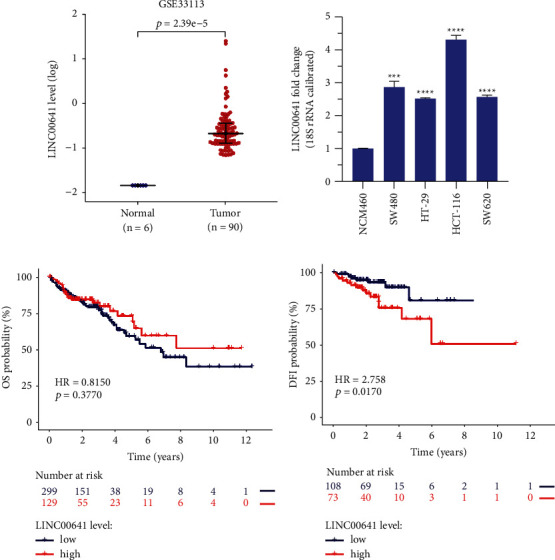
LINC00641 was upmodulated in CRC and highly expressed LINC00641 led to dismal clinical outcome (a) The GEO database revealed LINC00641 expression in colorectal cancer tissues. (b) qRT-PCR assessment of LINC00641 contents in normal human colon mucosal epithelial cell lines and CRC cell lines. Data are given as mean ± standard deviation (SD) of three biological replicates. (c) High expression of LINC00641 had no significant effect on overall survival (OS) by GDC database. (d) Highly expressed LINC00641 in human CRC tissues results in poor clinical outcome in disease-free interval (DFI) by UCSC Xena database. Relative to NCM460 cell, ^*∗∗∗*^*P* < 0.001, ^*∗∗∗∗*^*P* < 0.0001.

To assess the difference in LINC00641 expression, we retrieved CRC and nonmalignant tissues from the GEO database. Data illustrated that LINC00641 were remarkably upmodulated in the 90 CRC tissues in contrast with the 6 nonmalignant tissues (Figure 1(a)). qRT-PCR was then adopted to assess the mRNA contents of LINC00641 in four CRC cell lines (SW480, HT-29, HCT-116, and SW620) and normal human colon mucosal epithelial cell line (NCM460). It was found that the contents of LINC00641 were remarkably increased in all CRC cell lines in contrast with NCM460 cells (Figure 1(b)). Subsequently, the TCGA-COAD database was used to assess the relationship of the LINC00641 contents with the survival time. Although LINC00641 expression level failed to predict the overall survival (OS) (Figure 1(c)), CRC subjects with higher level of LINC00641 tend to have disease-free interval (DFI; Figure 1(d)). Altogether, these data indicate that LINC00641 was upmodulated in CRC and its expression was inversely linked to the patients' survival.

### 3.2. Knockdown of LINC00641 Inhibited CRC Cell Malignant Activities *In Vitro*

**Figure 2:**
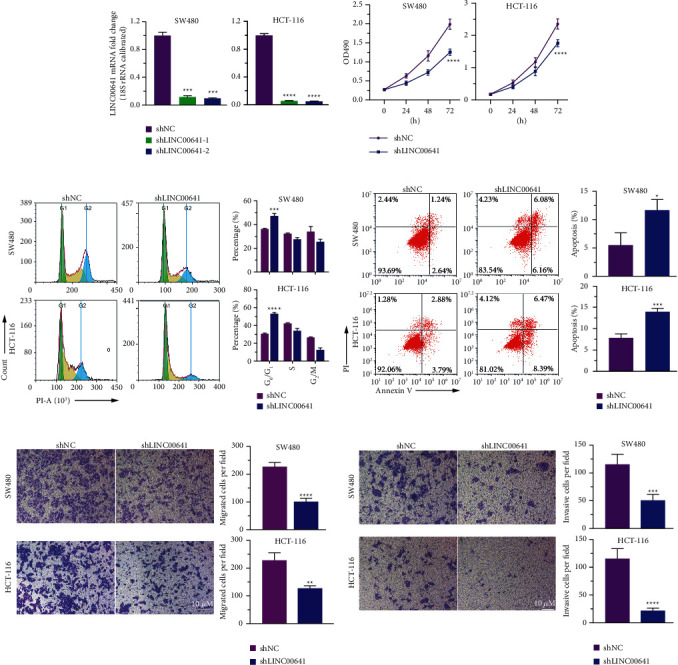
Silencing LINC00641 suppressed the malignant behavior of CRC cells *in vitro*. (a) qRT-PCR determining the knockdown efficiency of shRNAs. (b) MTT assay was used to investigate the effect of LINC00641 on SW480 and HCT-116 proliferation. (c), (d) The effect of LINC00641 on cell cycle and apoptosis was determined by flow cytometry. (e), (f) Silencing LINC00641 inhibited the migration and invasion ability of SW480 and HCT-116 cells. Data are represented as mean ± SD of three biological replicates. Compared with shNC group, ^*∗*^*P* < 0.05, ^*∗∗*^*P* < 0.01, ^*∗∗∗*^*P* < 0.001, ^*∗∗∗∗*^*P* < 0.0001. Scale bar: 10 *μ*m.

To evaluate the biological function of LINC00641 in CRC, endogenous LINC00641 was silenced in SW480 and HCT-116 cells by a lentivirus-based antagomir expression platform. The qRT-PCR analysis illustrated that LINC00641 expression was reduced by shLINC00641-1 and shLINC00641-2 compared to the control group (Figure 2(a)). Furthermore, the MTT assay and flow cytometry revealed that the knockdown of LINC00641 repressed cell proliferation (Figure 2(b)), G1/S phase transition in cell cycle (Figure 2(c)), and enhanced apoptosis (Figure 2(d)). Besides, transwell assays exhibited that silencing of LINC00641 in SW480 and HCT-116 cells remarkably dampened cell migration (Figure 2(e)) and infiltration (Figure 2(f)) *in vitro*. These results suggest that the knockdown of LINC00641 inhibits the growth, migration, and infiltration of CRC cells and promotes apoptosis of CRC cells *in vitro*.

### 3.3. miR-450b-5p Was a Direct Target of LINC00641

**Figure 3:**
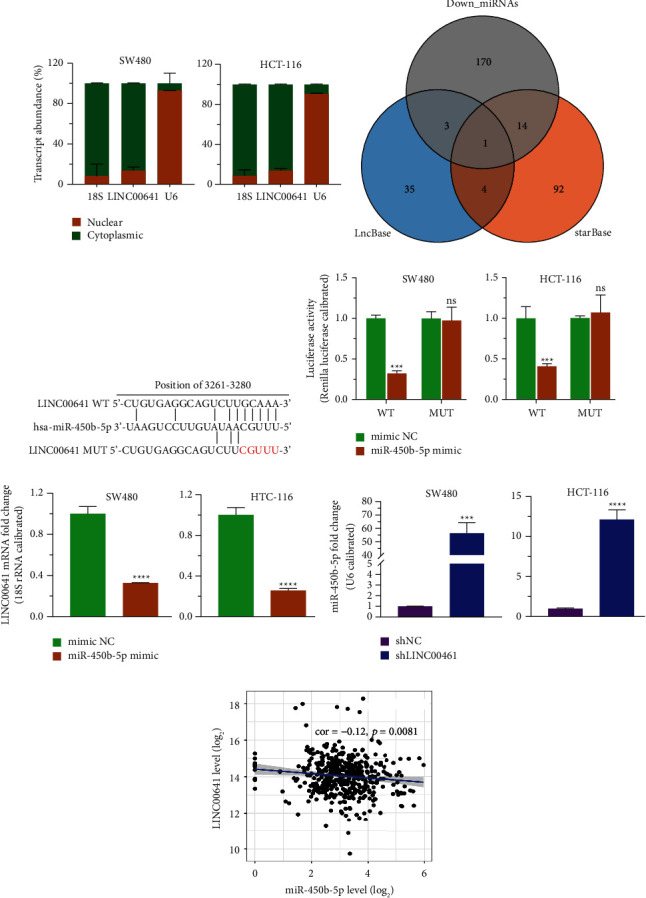
Cytoplasmic LINC00641 targeted miR-450b-5p in CRC cells (a) LINC00641 mainly existed in the cytoplasm, which was determined by subcellular fraction assay. (b) Venn diagram illustrating the overlap of LINC00641-targeting miRNAs in databases starBase and LncBase and downregulated miRNAs in GSE81581 dataset from GEO database. (c) At the complementary locations for the seed region in miR-450b-5p, mutations were introduced into the LINC00641 sequence. (d) The activities of the WT and mutant of LINC00641 were tested by luciferase reporter assay when has-miR-450b-5p was upregulated. (e) qRT-PCR analysis of the mRNA level of LINC00641 in SW480-NC, SW480- miR-450b-5p, HCT-116-NC, and HCT-116- miR-450b-5p cell lines. (f) qRT-PCR analysis of the level of miR-450b-5p in SW480-shNC, SW480-sh LINC00641, HCT-116-shNC, and HCT-116-shLINC00641 cell lines. (g) The correlation between miR-450b-5p and LINC00641 expression in CRC tissues. Compared with shNC group or mimic NC group, ^*∗∗∗*^*P* < 0.001, ^*∗∗∗∗*^*P* < 0.0001, ns *P* > 0.05.

Given that lncRNAs' biological roles were dependent on their cellular distribution, [23], we first explored the localization of LINC00641. On the basis of the subcellular fractionation assay, LINC00641 was primarily located in the cytoplasm (Figure 3(a)), which was further confirmed by *in silico* prediction data from an online database (Figure S1) and fluorescent in situ hybridization (FISH; Figure S2). Because cytoplasmic lncRNAs commonly work as ceRNAs to modulate the levels of target miRNAs [24], we checked the potential miRNAs targeted by LINC00641. After overlapping the miRNAs downregulated in the GSE81581 dataset with miRNAs that were predicted as candidate targets of LINC00641 in LncBase and starBase databases, only miR-450b-5p was enriched (Figure 3(b)). To examine whether miR-450b-5p serves as the valid target of LINC00641, we generated luciferase enzyme reporter vectors containing the wild-type (WT) or mutant (MUT) of LINC00641 (Figure 3(c)) to examine whether miR-450b-5p was the valid target of LINC00641. Results revealed that the luciferase enzyme activities of LINC00641-WT, but not LINC00641-MUT, were significantly decreased in SW480 and HCT-116 cells upon overexpression of miR-450b-5p (Figure 3(d)). qRT-PCR data exhibited that the LINC00641 mRNA contents were remarkably decreased in SW480/miR-450b-5p cells and HCT-116/miR-450b-5p compared to SW480/ctrl and HCT-116/ctrl cells (Figure 3(e)). Meanwhile, qRT-PCR illustrated that the contents of miR-450b-5p were remarkably increased by the knockdown of LINC00641 in both SW480 and HCT-116 cells in contrast with the controls (Figure 3(f)). In addition, the contents of miR-450b-5p in CRC tissues were inversely linked to the contents of LINC00641 in the CRC tissues (Figure 3(g)). Collectively, these data revealed that cytoplasmic LINC00641 targeted miR-450b-5p and negatively regulated its level.

### 3.4. miR-450b-5p Dampened the Growth, Migration, and Infiltration of CRC Cells *In Vitro*

**Figure 4:**
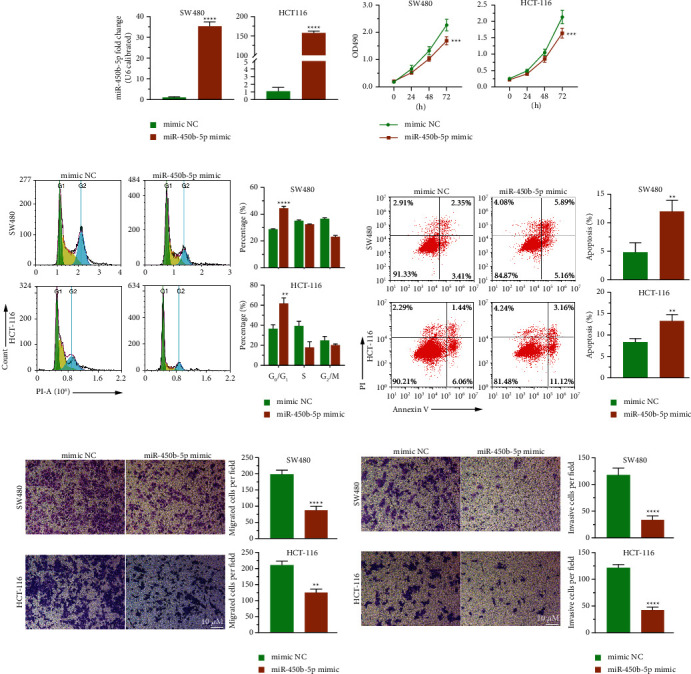
miR-450b-5p overexpression inhibited the malignant behavior of CRC cells *in vitro.* (a) qRT-PCR analysis showed that miR-450b-5p overexpression in the transfected SW480 and HTC-116 cell lines. (b) The proliferation of SW480 and HCT-116 was determined by MTT assay when miR-450b-5p was increased. (c), (d) The effect of miR-450b-5p on cell cycle and apoptosis was determined by flow cytometry. (e), (f) Ectopic expression of miR-450b-5p inhibited the migration and invasion ability of SW480 and HCT-116 cells, which was detected using transwell assay. Data are represented as mean ± SD of three biological replicates. Compared with mimic NC group, ^*∗∗*^*P* < 0.01, ^*∗∗∗*^*P* < 0.001, ^*∗∗∗∗*^*P* < 0.0001. Scale bar: 10 *μ*m.

Next, the biological function of miR-450b-5p in CRC was explored. Both the SW480 and HCT-116 cell lines demonstrated a significant reduction in cell proliferation when miR-450b-5p was expressed ectopically (Figures 4(a) and 4(b)). Increasing miR-450b-5p repressed cell G1/S phase transition (Figure 4(c)) and facilitated apoptosis (Figure 4(d)), which was determined by flow cytometry analysis. Moreover, SW480 and HCT-116 cell migration (Figure 4(e)) and invasion (Figure 4(f)) was significantly reduced *in vitro* when miR-450b-5p was overexpressed, which was demonstrated by transwell experiments. Overall, miR-450b-5p suppressed the malignant activities of CRC cells and promoted apoptosis.

### 3.5. GOLPH3 Was a Direct Target of miR-450b-5p

**Figure 5:**
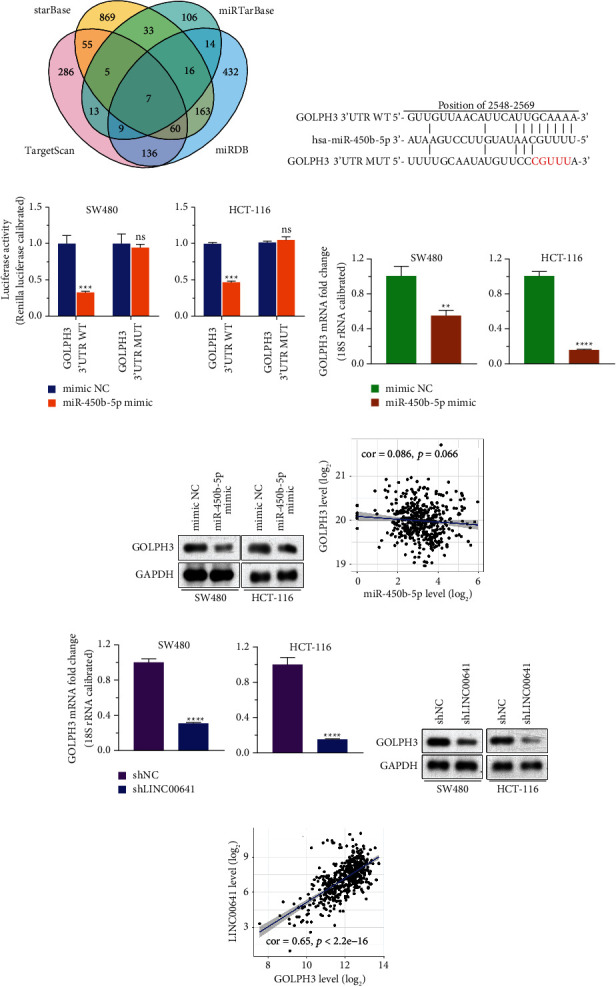
GOLPH3 was the downstream miR-450b-5p target. (a) Venn diagram illustrated the overlap of mRNAs in databases TargetScan, starBase, miRDB, and miRTarBase and predicted the putative downstream targets of miR-450b-5p. (b) GOLPH3's 3′UTR sequence was mutated at the corresponding locations for the seed region in miR-450b-5p. (c) The effects of miR-450b-5p expression on the activities of the WT and mutant 3′UTR of GOLPH3 in SW480 and HCT-116 cells were shown in a bar chart of Luciferase reporter assay analysis. (d) qRT-PCR analysis of the mRNA level of GOLPH3 in SW480-NC, SW480-miR-450b-5p, HCT-116-NC, and HCT-116- miR-450b-5p cell lines. (e) Western blot analysis showed the level of GOLPH3 proteins in SW480-NC, SW480-miR-450b-5p, HTC-116-NC, and HCT-116-miR-450b-5p cell lines. (f) The correlation between miR-450b-5p and GOLPH3 expression in CRC tissues was analyzed by starBase. (g) qRT-PCR analysis of the mRNA level of GOLPH3 in SW480-shNC, SW480-sh LINC00641, HTC-116-shNC, and HCT-116- shLINC00641 cell lines. (h) Representative image of western blotting showed the level of GOLPH3 proteins in SW480-shNC/shLINC00641 and HCT-116-shNC/shLINC00641 cell lines. (i) starBase analyzed the correlation between LINC00641 and GOLPH3 expression in CRC tissues. Compared with mimic NC group, ^*∗∗*^*P* < 0.01^*∗∗∗*^*P* < 0.001, ^*∗∗∗∗*^*P* < 0.0001, ns *P* > 0.05.

To explore further the underlying mechanism of miR-450b-5p in CRC, we explored the downstream targets of miR-450b-5p using four *in silico algorithms* (TargetScan, starBase, miRDB, and miRTarBase) (Figure 5(a)). Seven potential targets, including SESN3, C16orf72, CDC42EP3, GLCCI1, GOLPH3, CALM1, and CALM2, were enriched. We have previously reported the biological function of GOLPH3 in CRC, thus we chose GOLPH3 for following study [14–18]. GOLPH3 was predicted to harbor one of the miR-450b-5p docking sites in the 3′UTR of GOLPH3 at the 2548–2569 position, and the seed was indicated in Figure 5(b). Next, we cloned luciferase reporter vector harboring wild-type (WT) or mutant (MUT) 3′UTR of GOLPH3. It should be noted that the luciferase activities of GOLPH3 were remarkably decreased in both SW480 and HCT-116 cells upon miR-450b-5p overexpression. However, there was no change in the luciferase activity of GOLPH3-MUT (Figure 5(c)). Next, qRT-PCR and western blotting were adopted to assess the mRNA and protein contents of GOLPH3, respectively. Data illustrated that the mRNA and protein contents of GOLPH3 were remarkably decreased in SW480/miR-450b-5p and HCT-116/miR-450b-5p cells compared to SW480/ctrl and HCT-116/ctrl cells (Figure 5(d) and 5(e)). Notably, similar expression trends in miR-450b-5p and GOLPH3 were observed in CRC tissues (Figure 5(f)). These data illustrate that GOLPH3 is a direct target of miR-450b-5p.

To evaluate the differential expression between LINC00641 and GOLPH3, qRT-PCR and western blotting were performed to analyze the mRNA and protein levels, respectively, in SW480/shLINC00641 and HCT-116/shLINC00641 cells. Results indicated that the mRNA and protein contents of GOLPH3 were remarkably decreased in SW480/shLINC00641 and HCT-116/shLINC00641 cells relative to SW480/shNC and HCT-116/shNC cells (Figures 5(g) and 5(h)). Strikingly, the contents of GOLPH3 in CRC tissues were positively linked to the contents of LINC00641 (Figure 5(i)). Overall, these results further confirmed that LINC00641 acted as a ceRNA to modulate GOLPH3 expression via competing for miR-450b-5p.

### 3.6. Overexpression of GOLPH3 Reversed the Repressive Effects of LINC00641 Silencing in CRC Cells

**Figure 6:**
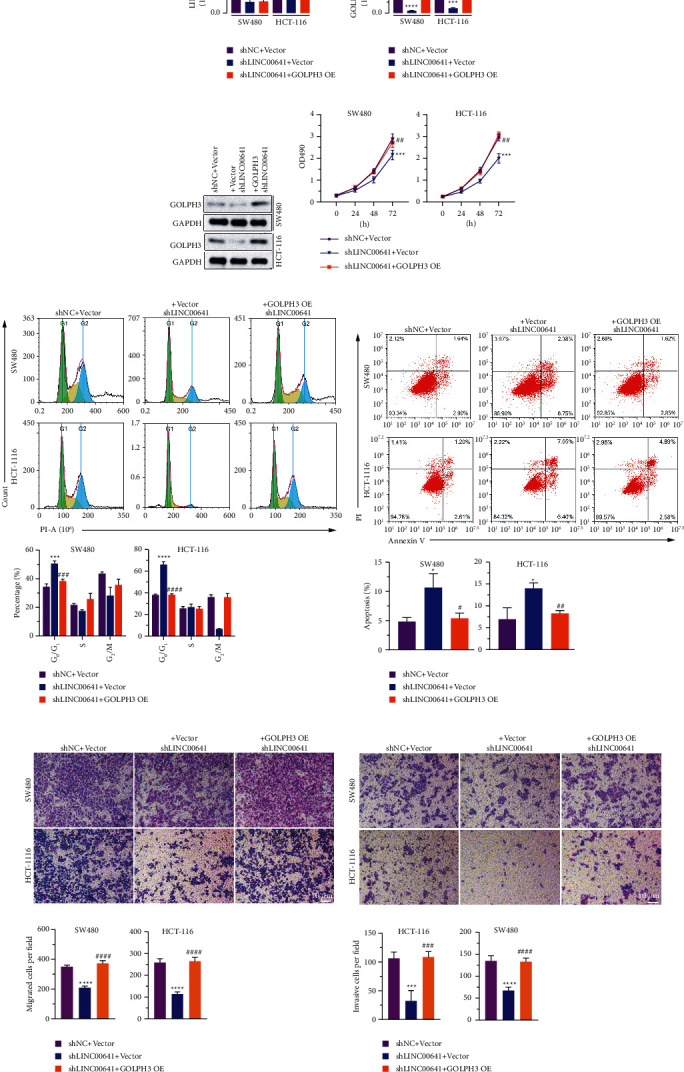
GOLPH3 overexpression partially reverted the impacts caused by LINC00641 silencing. (a)-(b) qRT-PCR analysis of LINC00641 and GOLPH3 expression in SW480/shNC + Vector, SW480/shLINC00641 + Vector, SW480/shLINC00641 + GOLPH3, HCT-116/shNC + Vector, HCT-116/shLINC00641 + Vector, and HCT-116/shLINC00641 + GOLPH3 cells. (c)Representative image of western blotting GOLPH3 expression in SW480/shNC + Vector, SW480/shLINC00641 + Vector, SW480/shLINC00641 + GOLPH3, HCT-116/shNC + Vector, HCT-116/shLINC00641 + Vector, and HCT-116/shLINC00641 + GOLPH3 cells. (d) MTT arrays indicated that GOLPH3 overexpression reversed the effect of LINC00641 silencing on proliferation. (e)-(f) Flow cytometry analysis showed that GOLPH3 overexpression reversed the effect of LINC00641 silencing on cell cycle and apoptosis. (g)-(h) Transwell assay showed GOLPH3 overexpression reversed the effect of LINC00641 silencing on migration and invasion. Data are represented as mean ± SD of three biological replicates. Compared with SW480/shNC + Vector or HCT-116/shNC + Vector, ^*∗*^*P* < 0.05, ^*∗∗∗*^*P* < 0.001, ^*∗∗∗∗*^*P* < 0.0001, ns *P* > 0.05. SW480/shLINC00641 + Vector vs. SW480/shLINC00641 + GOLPH3 OE, HCT-116/shLINC00641 + Vector vs. HCT-116/shLINC00641 + GOLPH3 OE, ^#^*P* < 0.05, ^##^*P* < 0.01, ^###^*P* < 0.001, ^####^*P* < 0.0001, ns *P* > 0.05. Scale bar: 10 *μ*m.

To determine whether GOLPH3 overexpression could reverse the function of LINC00641 in SW480 and HCT-116 cells, we measured GOLPH3 re-expression in SW480/shLINC00641 and HCT-116/shLINC00641 cell lines (Figure 6(a)). It was found that GOLPH3 expression was upmodulated in SW480 and HCT-116 cells co-transfected with GOLPH3 OE and shLINC00641 compared to cells co-transfected with vector and shLINC00641 (Figures 6(b) and 6(c)). The overexpression of GOLPH3 remarkably enhanced cell growth, cell cycle G1/S phase transition, migration, and infiltration and repressed cell apoptosis compared to the knockdown of LINC00641 (Figures 6(d)–6(h)). This suggests that the re-expression of GOLPH3 in SW480/shLINC00641 and HCT-116/shLINC00641 cells reverted the inhibitory impacts of LINC00641 silencing in CRC cells.

### 3.7. Knockdown of LINC00641 Dampened Tumor Growth *In Vivo*

**Figure 7:**
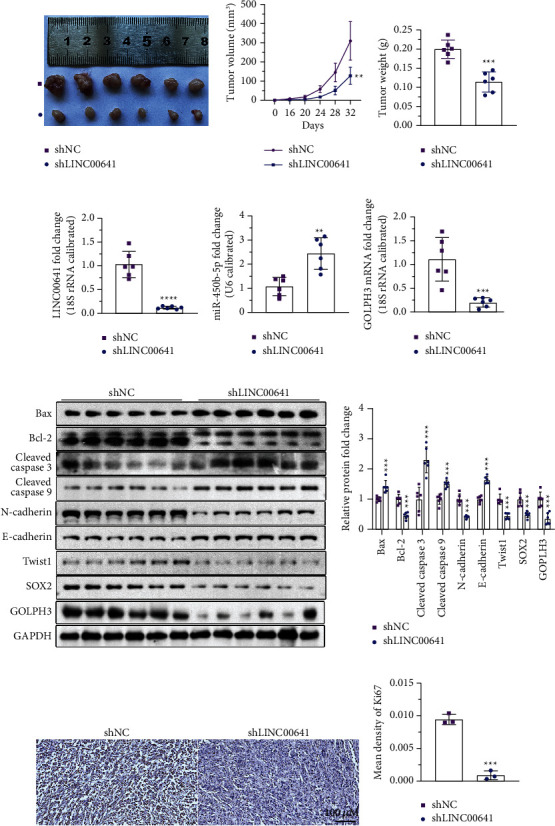
Knockdown of LINC00641 promoted EMT, stemness, and tumor growth *in vivo*. (a–c) The tumor weight and volume were repressed in the shLINC00641 group in contrast with the shNC group. (d–f) qRT-PCR assessment of the level of LINC00641, miR-450b-5p, and GOLPH3 in xenograft tumors. (g, h) Western blotting analysis and quantification of the protein levels. (i), j) Ki-67 immunohistochemical staining. Compared with shNC group, ^*∗∗*^*P* < 0.01, ^*∗∗∗*^*P* < 0.001, ^*∗∗∗∗*^*P* < 0.0001.

To explore whether the knockdown of LINC00641 could suppress the tumor growth of CRC cells *in vivo*, 5 × 10^6^ SW480/shLINC00641 and control cells were inoculated into the orthotopic sites of nude mice, respectively. As the results shown in Figure 7(a), LINC00641 knockdown dramatically suppressed tumor growth. A dramatic decrease in tumor volume and weight was reported in group shLINC00641 (Figures 7(b) and 7(c)). The downmodulation of LINC00641 was verified in the tumor tissues (Figure 7(d)), which was accompanied with an elevated content of miR-450b-5p and a reduced level of GOLPH3 (Figures 7(e)–7(h)). Moreover, the protein contents of Bax, cleaved caspase 3, and cleaved caspase 9 were elevated in group shLINC00641, contrary to the Bcl-2 protein level, which indicated that LINC00641 knockdown promoted cell apoptosis (Figures 7(g) and 7(h)). LINC00641 also modulated the protein contents of EMT markers consisting of N-cadherin and E-cadherin (Figures 7(g) and 7(h)). Besides, the contents of stemness genes SOX2 and Twist1 were determined. The data illustrated that LINC00641 deficiency reduced the protein levels of SOX2 and Twist1 (Figures 7(g) and 7(h)). Immunohistochemical data illustrated that the contents of Ki-67 were remarkably decreased after LINC00641 silencing (Figures 7(i) and 7(j)). Altogether, these data suggest LINC00641 promotes the tumorigenesis of CRC *in vivo*.

## 4. Discussion

To date, the effect of advanced CRC treatment is still unsatisfactory, despite the rapid development of technologies and therapeutic strategies. Thus, understanding the molecular mechanisms underlying CRC progress and metastasis is critical. Growing research evidence illustrates that lncRNAs play a remarkable role in regulating gene expression, cell signaling cascades, and chemoresistance in CRC [25–27]. Studies have reported that LINC00641 exerts the opposite effect in the progression of numerous types of cancer largely due to targeting different miRNAs. For example, LINC00641 was found to enhance the ability of cell growth and infiltration through sponging miR-340-5p in renal cell carcinoma [10], miR-429 in gastric cancer [11], and miR-378a in acute myeloid leukemia [28]. However, it has also been shown to suppress tumor growth and migration capacities via interacting with miR-365a-3p in prostate cancer [29], miR-4262 in glioma [30], miR-424 in cutaneous squamous cell cancer [31], miR-194-5p in breast cancer [32], miR-424-5p in NSCLC [9], and miR-197-3p in bladder cancer [33]. Herein, results indicated that LINC00641 contents were correlated with dismal prognosis in CRC, and LINC00641 silencing remarkably dampened growth and infiltration of colon cancer cells *in vitro* and *in vivo*. Additionally, it was revealed that LINC00641 promotes EMT and CSC stemness *in vivo* assay. Therefore, LINC00641 shows pivotal effects in the promotion of tumor in CRC.

Several investigations have documented that lncRNAs can sequester miRNAs through acting as ceRNAs or as RNA sponges [5, 34]. MicroRNAs (miRNAs) constitute endogenous small 18–25 nucleotide noncoding RNA molecules that attach to the 3′UTR of particular target mRNAs, causing mRNA degradation and translation. Evidence exhibits that miRNAs harbor remarkable roles in the onset and progress of numerous diseases, including cancer. In this context, we utilized bioinformatics to uncover miRNAs that can cross talk with LINC00641. Subsequently, miR-450b-5p, which had the highest binding score, was chosen to perform further experiments. In various forms of human malignancies, such as rhabdomyosarcoma, CRC, hepatocellular cancer, nasopharyngeal carcinoma, and breast cancer, miR-450b-5p is downmodulated and functions as a tumor repressor [22, 35–39]. Herein, we established that miR-450b-5p dampens the growth, migration, and infiltration of CRC cells *in vitro*, which suggests that miR-450b-5p works as a tumor repressor in CRC. A previous study showed miR-450b-5p inhibits stemness and progress of chemoresistance to 5-FU via targeting SOX2 in CRC [22]. Herein, the luciferase reporter gene validated the targeting relationship of LINC00641 with miR-450b-5p. And, *in vivo* assay, LINC00641 also regulated the protein contents of EMT markers, for instance N-cadherin and E-cadherin. The data illustrated that LINC00641 deficiency reduced the protein levels of SOX2 and Twist1. This indicated that LINC00641 enhances EMT and CSC stemness through acting as a sponge of miR-450b-5p in CRC.

Furthermore, the 3′UTR of GOLPH3 has been discovered to contain highly conserved miR-450b-5p binding sites. Recent studies have confirmed that the GOLPH3 gene plays the role of an oncogene, and upregulation of GOLPH3 expression promoted colon cancer cell growth, invasion, and chemoresistance [16, 40–42]. Herein, the luciferase enzyme reporter assay verified that GOLPH3 is the downstream target of miR-450b-5p. Moreover, GOLPH3 was positively regulated by LINC00641, but was inversely linked to the expression of miR-450b-5p. Moreover, the rescue assays proved that the overexpression of GOLPH3 reversed LINC00641 silencing-mediated proliferation, apoptosis, migration, and infiltration in CRC cells. Thus, we concluded that LINC00641 acts as a ceRNA in CRC via sponging miR-450b-5p to upmodulate GOLPH3. In addition, the cross talk of LINC00641 with miR-450b-5p upregulated GOLPH3 expression, ultimately contributing to the progress of CRC.

This research has some limitations. For example, we did not directly detect the LINC00641 levels in human tissues because of lack of clinical samples. We are seeking for Ethics Committee approval to collect clinical samples for LINC00641 detection using qRT-PCR. Additionally, we will also purchase tissue microarrays for LINC00641 detection using FISH after the COVID-19 pandemic, which is breaking out in Quanzhou City now. In addition, SW480 cells containing luciferase vector should be used to construct *in situ* xenograft and metastatic models to elucidate the effect of LINC00641 on CRC progression.

## 5. Conclusions

In summary, we established that LINC00641 was upmodulated in CRC and was associated with dismal prognosis. Results revealed that it played the function of an oncogene and upregulated GOLPH3 expression through sponging miR-450b-5p in colon cancer. Overall, our results suggested that LINC00641/miR-450b-5p/GOLPH3 axis might serve as a novel therapeutic target for CRC.

## Data Availability

The analyzed datasets generated during this work are accessible upon request from the corresponding author.

## Supplementary Materials

Supplementary Materials Figure S1. Lnclocator (http://www.csbio.sjtu.edu.cn/bioinf/lncLocator/) predicted the nucleocytoplasmic localization of lncRNA LINC00641, and the prediction data illustrated that it was in the cytoplasm. Figure S2. Images of Fluorescence in situ hybridization (FISH) illustrating the location of LINC00641. Table S1. Primer sequences for qRT-PCR. Table S2. Primer sequences of plasmid constructs.
