# Supplementary figures and images for: Long Intergenic Noncoding RNA 00641 Promotes Growth and Invasion of Colorectal Cancer through Regulating miR-450b-5p/GOLPH3 Axis
Source: J Oncol. 2022 Jun 15;2022:8259135. doi: 10.1155/2022/8259135 (PMC9217543; doi:10.1155/2022/8259135)

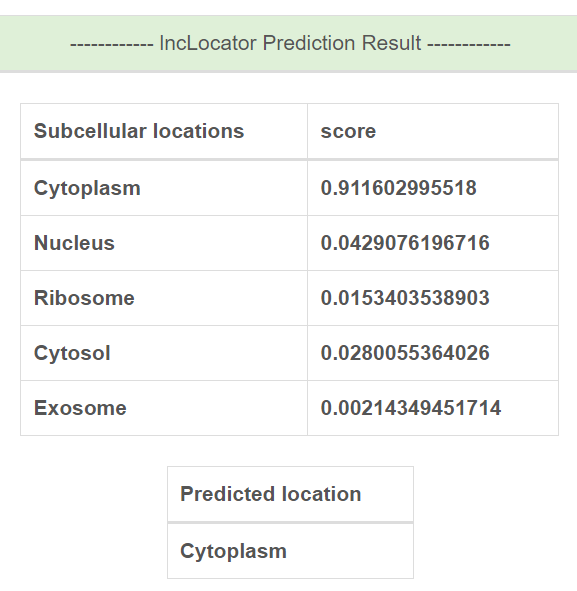

Supplement: Supplementary Materials — Figure S1. Lnclocator (http://www.csbio.sjtu.edu.cn/bioinf/lncLocator/) predicted the nucleocytoplasmic localization of lncRNA LINC00641, and the prediction data illustrated that it was in the cytoplasm. Figure S2. Images of Fluorescence in situ hybridization (FISH) illustrating the location of LINC00641. Table S1. Primer sequences for qRT-PCR. Table S2. Primer sequences of plasmid constructs. [file 8259135.f1.zip › 8259135.f1/Figure S1.png]

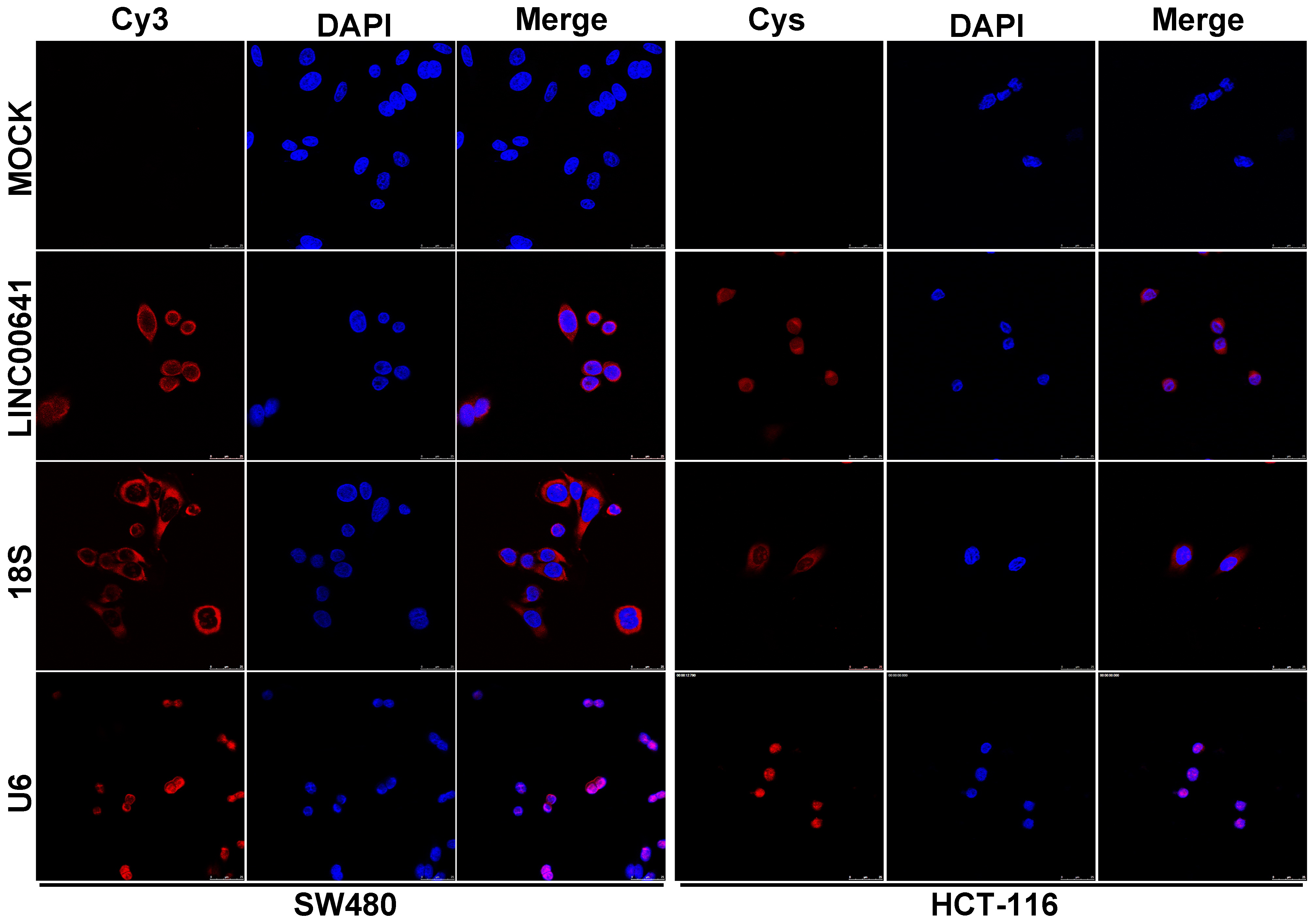

Supplement: Supplementary Materials — Figure S1. Lnclocator (http://www.csbio.sjtu.edu.cn/bioinf/lncLocator/) predicted the nucleocytoplasmic localization of lncRNA LINC00641, and the prediction data illustrated that it was in the cytoplasm. Figure S2. Images of Fluorescence in situ hybridization (FISH) illustrating the location of LINC00641. Table S1. Primer sequences for qRT-PCR. Table S2. Primer sequences of plasmid constructs. [file 8259135.f1.zip › 8259135.f1/Figure S2.jpg]
